# Knock-Down of CD44 Regulates Endothelial Cell Differentiation via NFκB-Mediated Chemokine Production

**DOI:** 10.1371/journal.pone.0090921

**Published:** 2014-03-10

**Authors:** Berit Olofsson, Helena Porsch, Paraskevi Heldin

**Affiliations:** Ludwig Institute for Cancer Research, Science for Life Laboratory, Uppsala University, Biomedical Center, Uppsala, Sweden; University of Patras, Greece

## Abstract

A striking feature of microvascular endothelial cells is their capacity to fuse and differentiate into tubular structures when grown in three-dimensional (3D) extracellular matrices, in collagen or Matrigel, mimicking the *in vivo* blood vessel formation. In this study we demonstrate that human telomerase-immortalised foreskin microvascular endothelial (TIME) cells express high levels of the hyaluronan receptor CD44 and the hyaluronidase HYAL2. Knock-down of CD44 or HYAL2 resulted in an inability of TIME cells to form a tubular network, suggesting a key regulatory role of hyaluronan in controlling TIME cell tubulogenesis in 3D matrices. Knock-down of CD44 resulted in an upregulation of mRNA expression of the chemokines CXCL9 and CXCL12, as well as their receptors CXCR3 and CXCR4. This was accompanied by a defect maturation of the tubular structure network and increased phosphorylation of the inhibitor of NFκB kinase (IKK) complex and thus translocation of NFκB into the nucleus and activation of chemokine targed genes. Furthermore, the interaction between CD44 and hyaluronan determines the adhesion of breast cancer cells. In summary, our observations support the notion that the interaction between CD44 and hyaluronan regulates microvascular endothelial cell tubulogenesis by affecting the expression of cytokines and their receptors, as well as breast cancer dissemination.

## Introduction

Endothelial cell morphogenesis which occurs during embryonal vasculogenesis and angiogenesis is based on the abilities of endothelial cells to migrate, proliferate, organize themselves into tubular structures, and to maintain the stability and maturation of neo-vessels [1], [2], [3]. The maintenance of vascular integrity is regulated by several mechanisms including cell-cell junctions and a glycocalyx around the endothelial cells [4], [5], [6]. The glycocalyx is a mesh of proteoglycans, glycolipids and glycosaminoglycans which is integrated with membrane adhesive proteins of endothelial cells [4].

The glycosaminoglycan hyaluronan is a prominent component of endothelial glycocalyx and has both structural and signaling roles [6]. Hyaluronan is synthesized by hyaluronan synthases (HAS1, HAS2, HAS3) [7], [8], [9] and degraded by hyaluronidases (HYAL1, HYAL2) [10], [11]. Ausprunk [12] demonstrated that during the formation of chorioallantoic membrane capillaries, hyaluronan-rich matrices rapidly disappeared most likely because of degradation by HYALs. Studies by us and other laboratories revealed that hyaluronan in a size-dependent manner affects the formation of vessel-like structures in 3D collagen or Matrigel cultures; hyaluronan fragments of 3–25 disaccharide units promote tube formation whereas high molecular mass hyaluronan suppresses tube formation [13], [14], [15], [16], [17], [18]. The molecular mechanisms underlying hyaluronan production in endothelium are not well understood, but pro-inflammatory stimuli such as TNFα and IL-1β as well as the vascular endothelial growth factors (VEGF) A and B, have been shown to induce the synthesis of hyaluronan in endothelial cells derived from microvasculature, but not from large vessels [19], [20].

Hyaluronan and hyaluronan fragments can modulate cell proliferation, migration and differentiation through interactions with specific cell surface receptors, the best characterized of which are CD44 and RHAMM [21], [22], [23], [24], [25], [26]. CD44 is a cell-surface glycoprotein which is expressed in multiple forms due to alternative splicing of 10 variable exons and subsequent post-translational modifications, such as glycosylation and addition of glycosaminoglycan chains [22], [24]. The most widely expressed CD44 is the standard form (CD44s) which is found on the surface of hematopoietic, epithelial, endothelial and mesenchymal cells. The variant isoforms, CD44 v1-10, are preferentially expressed in epithelial malignancies [27]. CD44 is involved in cell-cell and cell-extracellular matrix interactions, for example through its interaction with the IQ motif containing GTPase activating protein (IQGAP)1 which plays a key regulatory role in cell-cell junctions [28]. Furthermore, CD44 has been shown to function as a co-receptor for cytokine and growth factor receptors, including the receptors for platelet-derived growth factor (PDGF), transforming growth factor β (TGFβ), epidermal growth factor (EGF) and hepatocyte growth factor (HGF). During such a cross-talk, hyaluronan-activated CD44 can modulate the response of cells to growth factors [29], [30], [31], [32], [33]. RHAMM was initially discovered as a soluble hyaluronan binding protein that is important in cell migration [34], but later the protein was also found on the cell surface and intracellularly. It is expressed preferentially at sites of tissue injury, inflammation and cancer [21], [35], [36]. CD44 and RHAMM can both signal through the Erk1/2 MAP kinase signaling pathway to regulate breast cancer motility, but also have different affects on cellular signaling [36], [37], [38], [39].

With regard to endothelial cell functions, CD44-hyaluronan fragment interactions elicit intracellular signals modulating cell proliferation, migration and tubular morphogenesis [15], [16], [17], [38], [40], [41]. Previously, we have demonstrated that one mechanism of the angiogenic action of hyaluronan fragments upon their binding to CD44 is the production of the chemokine CXCL1 and subsequent activation of its receptor CXCR2 [17]. CXCL1 mediates pro- and anti-angiogenic functions in addition to triggering inflammation, stem cell survival and homeostasis [42], [43]. Furthermore, hyaluronan sequestrated on endothelial surface binds to CD44 expressed on lymphocytes and contributes to extravasation of circulating lymphocytes at sites of inflammation [19] and regulates vascular permeability [6]. In addition, a CD44-dependent adhesion of a leukemic cell line to the endothelium has been reported [44]. The mechanisms whereby CD44 and hyaluronan fragments affect angiogenesis, tumor cell dissemination and homeostasis are yet not known. In this study, we demonstrate that microvascular endothelial cells express high levels of CD44 and HYAL2 and investigated their functional roles in cotrolling the formation of vessel-like structures and dissemination of breast cancer cells.

## Materials and Methods

### Cell culture

Human telomerase-immortalised foreskin microvascular endothelial (TIME) cells were generously provided by Dr. L. Claesson-Welsh (gift from Dr M. McMahon, UCSF, University of California, USA) [45]. The cells were routinely cultured under proliferating conditions on plastic dishes coated with 0.25% gelatin and maintained in endothelial growth medium (EBM-2, PromoCell), supplemented with 10% fetal calf serum (FCS), 5 ng/ml epidermal growth factor (EGF), 0.5 ng/ml VEGF, 10 ng/ml fibroblast growth factor 2, 20 ng/ml insulin-like growth factor 1, 1 µg/ml ascorbic acid and 0.2 µg/ml hydrocortisone. In this study, cells between passages 20 and 30 were used. In order to study the cells under differentiating conditions, endothelial cells were grown on collagen type I gels (Vitrogen) or Matrigel (BD Biosciences).

The aggressive human breast cancer cell line MDA-MB-231 was kindly provided by Dr. J. Bergh (Karolinska Institute, Stockholm, Sweden). A clone of this cell line which forms bone metastases (MDA-MB-231-BM cells which are labelled with GFP) was generously provided by Dr. P. ten Dijke (University of Leiden, The Netherlands). Breast cancer cells were routinely maintained in DMEM (Gibco) supplemented with 10% FCS (Biowest), penicillin (100 µg/ml) and streptomycin (100 µg/ml; SVA Uppsala, Sweden).

### RNA isolation and real time PCR

Total RNA was extracted from TIME cells, cultured under proliferative or differentiating conditions, using the RNeasy Mini kit (Qiagen) according to the manufacturer's instructions and reverse-transcribed to cDNA using the iScript cDNA synthesis kit (Biorad). Real time PCR was carried out on a Biorad bcfx96 cycler using iQ SYBR Green Supermix (Biorad) according to the manufacturer's instructions. Primer sequences for HAS1, HAS2, HAS3, HYAL1, HYAL2, CD44s, CD44v3, CD44v6 and GAPDH were published previously [46]. The primers for chemokines CXCL9, CXCL12 and their receptors CXCR3, CXCR4, respectively, as well as for IL-6 and the adhesion receptors ICAM-1 and VCAM-1were designed using the NCBI website (the specific sequences are shown on Table 1). The expression level of each target gene was normalized to the endogenous reference gene, GAPDH, and was calculated as 2^−ΔCT^×100 (ΔCT = CT (sample mRNA)−CT (GAPDH mRNA)).

**Table 1:** Primer Sequences.

Primer	Sequence
CXCL9-F	5′-CCAGTAGTGAGAAAGGGTCGC-3′
CXCL9-R	5′-AGGGCTTGGGGCAAATTGTT-3′
CXCL12-F	5′-GGACTTTCCGCTAGACCCAC-3′
CXCL12-R	5′-GTCCTCATGGTTAAGGCCCC-3′
CXCR3-F	5′-GTCCTTGAGGTGAGTGACCA-3′
CXCR3-R	5′-AGCACGAGTCACTCTCGTTT-3′
CXCR4-F	5′-GCAGCAGGTAGCAAAGTGAC-3′
CXCR4-R	5′-GCCCATTTCCTCGGTGTAGT-3′
CD34-F	5′-TACACGGAAAACGGTGGAGG-3′
CD34-R	5′- TTTTCTGAGCCCCTCGGTTC-3′
ICAM-1-F	5′-ACTGACCCCAACCCTTGATG-3′
ICAM1-R	5′-GGTGACCTTGAATGTGACATGG-3′
VCAM-1-F	5′-CAATTCACATGGCATAGTCGTT-3′
VCAM-1-R	5′-CCAATGTGGGTTAAGGGGGT-3′
IL-6-F	5′-CTTCGGTCCAGTTGCCTTCT-3′
IL6-R	5′-TGGAATCTTCTCCTGGGGGT-3′

Primer sequences used for the quantification of gene expression, using real time PCR, in human dermal microvascular endothelial cells.

### siRNA transfection

TIME cells were transiently transfected with 5 nM of siRNAs for scrambled control, HYAL2 or CD44 for 24 h, followed by subculture on plastic dish or on Matrigel for another 16 h. In some experiments, prior to seeding them on Matrigel, cells were pre-treated for 1 h with 36 µM of the cell-permeable NF-kB SN50 inhibitor peptide or the control inactive peptide SN50M (both from Calbiochem). All siRNAs were purchased from Dharmacon (ONTarget SMARTpool Plus) and transfected into the cells using SilentFect reagent (Biorad) according to the manufacturer's instructions. Knockdown efficiency was routinely checked at the mRNA and/or protein levels.

### Detection of hyaluronan in the media

Conditioned media of cultures expressing HYAL2 and CD44 or not, were collected and the hyaluronan content was quantified using a competitive binding assay [47]. For analysis of the endogenous hyaluronidase HYAL2 activity, 800 ng/ml hyaluronan (high molecular weight, Q-Med, Uppsala, Sweden) per 1×10^6^ cells transiently transfected with siRNA for scrambled control, HYAL2 and CD44 was added, and the cultures were grown for 24 h. Thereafter, the hyaluronan content in conditioned media was analyzed.

### Adhesion assay

TIME cells (6×10^4^ cells/well) were grown in 24-well plates, pre-coated with 0.25% gelatine, overnight at 37°C and 5% CO_2_ to confluency. To evaluate the role of CD44 expressed by TIME cells as an anchor in dissemination of breast cancer cells surrounded by hyaluronan-rich pericellular matrices, TIME cells were pre-treated with CD44-blocking antibody Hermes-1 or rat IgG, each of 5 µg/ml, for 30 min at 37°C. The human breast cancer cell line MDA-MB-231 and a clone of this cell line that forms bone metastases MDA-MB-231-BM [46] were used. Breast cancer cells were gently detached in PBS supplemented with 10 mM EDTA, pre-treated with 16 U/ml *Streptomyces* hyaluronidase (or PBS as a control) at 37°C for 30 and their viability checked with trypan blue. Then, 5×10^4^ breast cancer cells per well were plated onto a confluent endothelial cell monolayer and allowed to adhere for up to 4 h. At the indicated time points, non-adherent breast cancer cells were removed with aspiration and gentle washing with PBS. Endothelial/tumour cell co-cultures were fixed in 2% paraformaldehyde, 0.2% glutaraldehyde at room temperature for 20 min and kept in PBS. Photos of five randomly chosen fields per well were taken with a Zeiss Axiovert40 phase-contrast microscope and the number of adherent cells was counted (triplicate wells).

### Tubulogenesis assay

To induce TIME cell differentiation and the formation of tubular-like structures, ice-cold 12-well plates were coated with 200 µl growth factor-reduced Matrigel (BD Biosciences) per well, resulting in a 1 mm thick gel. The gels were allowed to solidify at 37°C for at least 2 h. Then, cells expressing or depleted of CD44 and HYAL2 using specific siRNAs, were trypsinized, counted and 2×10^5^ cells were seeded on Matrigel-containing plates. Tubular morphogenesis proceeded for the indicated time periods and phase contrast photographs were taken with a Zeiss Axiovert40 microscope. Tubular-like structures were isolated by Dispase treatment (50 U/ml, BD Biosciences); 400 µl of Dispase solution was added for 1 h at 37°C to digest the Matrigel. The reaction was stopped by addition of 800 µl of 10 mM EDTA in PBS followed by centrifugation and washing in PBS. Cell pellets were used for RNA extraction or protein detection using Western blotting.

TIME cells (1×10^5^ cells) were seeded on 250 µl collagen gel layers per well into 24-well plates for up to 16 h, essentially as described previously [17]. Then, cells were fixed in 3% paraformaldehyde for 15 min, permeabilised with 0.2% Triton X-100 and non-specific binding was blocked in 20% goat serum. Cells were stained with anti-CD31 (1∶100, Dako) or anti-CD44 (Hermes 1, 1 µg/ml) in 4% goat serum. Alexa Fluor 568-labelled goat anti-rat and Alexa Fluor 488-labelled goat anti-mouse antibodies (Molecular Probes) were used both at a concentration of 1∶1000 in 1% BSA in PBS. The nuclei were stained with DAPI and the slides were mounted with ProLong gold antifade reagent (Invitrogen). Photographs were taken with a Zeiss Axioplan 2 immunofluorescence microscope using Volocity software.

### Immunoblotting and immunoprecipitation

TIME cells grown on plastic dish were lysed in ice-cold 20 mM Tris, 137 mM NaCl, 5 mM EDTA, 1% Triton X-100, pH 7.9, supplemented with protease inhibitors and incubated on ice for 30 min, sheared through a 20 gauge needle, re-incubated on ice for another 30 min and centrifuged at 13000 rpm at 4°C for 15 min. Cells cultured onto Matrigel prior lysis were subjected to Dispase digestion as described above. Protein concentration in the supernatant was determined using the BCA kit (Pierce). The proteins were separated by sodium dodecyl sulphate-polyacrylamide gel electrophoresis (SDS-PAGE) using 10% polyacrylamide gels, transferred to a nitrocellulose membrane, blocked in 5% milk or 5% BSA in 20 mM Tris, 137 mM NaCl, 1% Tween-20, pH 7.9 (TBS-T), and immunoblotted using antibodies against CD44 (Hermes3, 1 µg/ml, generously provided by Dr S. Jalkanen, Turku, Finland), β-actin (1∶10000, Sigma), CD31 (1∶100, Dako), HYAL2 (1 µg/ml, [32]), p-FAK Y397 and total FAK (1∶1000, BD Biosciences), total Akt 1/2/3 (1∶250, Santa Cruz), p-Akt S473 and total Erk1/2 (1∶500, Cell Signaling), p-STAT3 and total STAT3, p-p38 and total p-38, p-Erk1/2, p-IKKα/β S176/180 and cleaved Caspase-3 (1∶1000, Cell Signalling) as well as total IKKα/β (1∶1000, Santa Cruz). Following washings in TBS-T, the membranes were incubated with the respective HRP-conjugated secondary antibodies and immunocomplexes were detected by chemoluminescence and Kodak X-ray films.

To detect the interaction between CD44 and HYAL2, after cell lysis samples were pre-cleared with 10 µl protein G-Sepharose beads (GE Healthcare; 50% slurry in PBS) end-over-end at 4°C for 1 h and then incubated with 3 µg primary antibody (Hermes 3 or HYAL2) or corresponding IgG isotype control (Santa Cruz) end-over-end at 4°C over-night. The immune-complexes were captured by 25 µl protein G-Sepharose beads with end-over-end mixing for 1 h at 4°C. Beads were washed four times by centrifugation (300× g, 5 min) in cell lysis buffer and then boiled at 95°C for 5 min in 20 µl reducing SDS-sample buffer inducing the elution of the captured proteins. Beads were removed by centrifugation (300× g, 5 min) and the supernatant was analyzed by SDS-PAGE and immunoblotting.

### PCR array

An angiogenesis-specific RT^2^ Profiler PCR array (PAHS-024Z, SABiosciences) was performed according to the instructions of the manufacturer. Briefly, the RNAs from CD44- or HYAL2-depleted cells, grown under differentiating conditions, were extracted using the RNeasy Mini kit (QIAGEN) including the on-column DNase digestion step (using RNase free DNase set, QIAGEN). Then, RNA (0.7 µg) was reversely transcribed to cDNA using the iScript Kit (Biorad) and quantitative real time PCR analysis was performed using iQ SYBR Green Mastermix (Biorad) and Biorad cfx96 cycler. Gene expression was compared to control and thresholds for up- and downregulation were set to 2 and 0.5, respectively.

## Results

### Microvascular endothelial cells express high levels of CD44s and HYAL2

**Figure 1:**
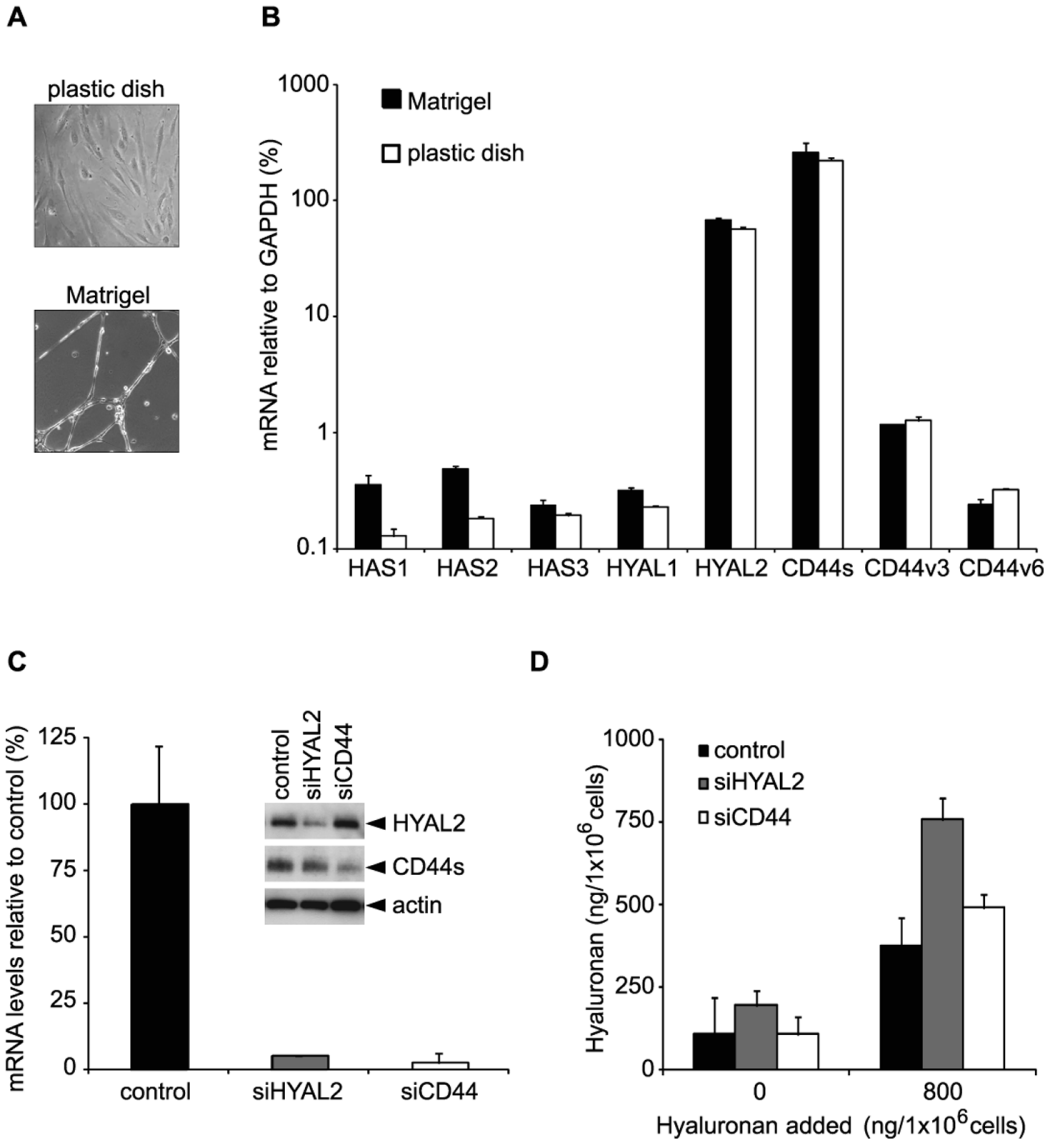
Characterisation of microvascular endothelial cells. *(A) Phase contrast microscopy of TIME cells cultured on plastic dish or in Matrigel*. TIME endothelial cells (2×10^5^) were grown in 12-well plates under proliferating conditions on solid surface, or under differentiating conditions in Matrigel, for 16 h. *(B) Expression of HAS, HYAL and CD44 under proliferating and differentiating conditions*. Expression levels relative to GAPDH of HAS1, 2, 3, HYAL1, 2 and CD44s, v3, v6 mRNAs were determined by real time PCR, as described in Materials and Methods. Results are mean of three separate experiments performed in triplicates ± S.D. *(C) Detection of knock-down of HYAL2 or CD44 at mRNA and protein level*. Endothelial cells were transfected with 5 nM of siRNA targeting either HYAL2 or CD44. RNA was extracted after 24 h and subjected to real time PCR to determine the knock-down efficiency at the mRNA level. For Western blotting, cells were lysed after 48 h of transfection and subjected to SDS-PAGE (insert). *(D) Hyaluronan production and hyaluronidase activity*. TIME cells were transfected with siRNAs (scrambled control, HYAL2 or CD44) for 24 h. Then, medium was changed to growth medium containing or not containing 800 ng hyaluronan/1 million cells. Conditioned media were collected after 24 h and the hyaluronan content detected, as described in Materials and Methods. A representative experiment, out of three separate experiments performed with similar results is shown.

During the course of an inflammatory response, hyaluronan is found at the luminal surface of blood vessels i.e. in the endothelial glycocalyx [6], [12], [19]. *In vitro*, microvascular endothelial cells cultured in growth medium on gelatin-coated dishes grow as a monolayer and proliferate, whereas when grown on Matrigel they differentiate and form tubular-like structures (Figure 1A). To examine the contribution of HASs, HYALs and CD44 for the presence of hyaluronan in the glycocalyx layer of microvascular endothelial cells, we investigated their expression levels under both proliferative and differentiating conditions. As shown in Figure 1B, real-time PCR analysis revealed that HAS1, HAS2 and HAS3 were expressed at about 10–20-fold higher level, when cells were grown on Matrigel compared to growth under proliferating conditions on solid surface culture dishes. The expression of HAS2 during microvascular endothelial cell morphogenesis was 2-fold and 3-fold higher than that of HAS1 and HAS3, respectively. HYAL2 mRNA was the predominantly expressed isoform among the HYALs (about 250-fold higher than the expression of HYAL1) under both proliferative and differentiating conditions. mRNA for the standard form of CD44 was expressed under both proliferative and differentiating conditions at about 220-fold and 1025-fold higher levels compared to the variant forms CD44v3 and CD44v6, respectively (Figure 1B).

### TIME cells express functionally active hyaluronidase

HYAL1 and HYAL2 are the major hyaluronidases in tissues and account for the fragmentation of hyaluronan in a concerted manner [48], [49]. The prominent expression of HYAL2 by ECs prompted us to investigate its involvement in the degradation of endogenous and exogenously added hyaluronan. A silencing of HYAL2 mRNA by 90% (Figure 1C) resulted in an about 2-fold increase in the amount of endogenous hyaluronan (Figure 1D). When exogenous hyaluronan was added to microvascular endothelial cultures expressing HYAL2, a substantial amount of it was degraded to sizes undetectable by the hyaluronan detection assay used; the assay is based on the specific and irreversible capture of hyaluronan molecules of a molecular mass higher than 100 000 with the hyaluronan binding protein domain of aggrecan (unpublished observation). However, knock-down of HYAL2 lead only to a minute decrease of the exogenously added hyaluronan (Figure 1D). This data indicate that HYAL2 is functionally active. The suppression of CD44 mRNA by siRNA to about 10% of the level of the scrambled control had no effect on the fragmentation of neither endogenous nor exogenously added hyaluronan (Figure 1C, 1D). The knockdown of CD44 did not affect the expression of HYAL2 and vice versa (Figure 1C, insert). Whereas a physical interaction between CD44 and HYAL2 has been demonstrated in breast cancer cell line MDA-MB-231 and rat v-Src-transformed fibroblasts [50], [51], we were unable to demonstrate such an interaction in TIME cells using co-immunoprecipitation (data not shown).

### CD44 expressed by TIME cells anchors to hyaluronan and determines breast cancer cell adhesion

**Figure 2:**
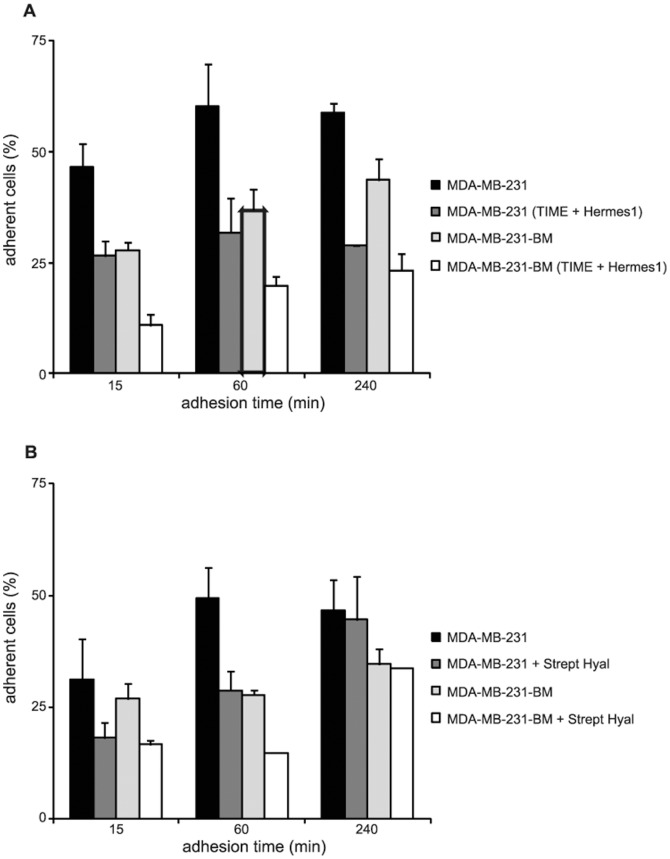
CD44 anchors hyaluronan to the surface of microvascular endothelial cells. *(A)* TIME cell monolayers were incubated for 30 min with CD44 blocking antibody, Hermes1, or control IgG, followed by media aspiration before the seeding of MDA-MB-231 and MDA-MB-231-BM cell lines. *(B)* The tumor cells were seeded on confluent cultures of TIME cells before or after pre-treated with *Streptomyces* hyaluronidase. The percentage of adherent cells was determined at the indicated time points. The average ± S.D. of triplicates from two separate experiments is shown.

Hyaluronan sequestration on endothelium is specialized to support lymphocyte adherence through interaction with lymphocyte CD44 [52]. Most likely, during dissemination to distant organs, cancer cells use similar strategies as leukocytes to overcome the shear forces of blood flow [53]. The predominant expression of CD44 by microvascular endothelial cells (Figure 1B) [15] prompted us to investigate its involvement in breast cancer transient adhesion to endothelium as the first step during their dissemination. The MDA-MB-231 and MDA-MB-231-BM breast cancer cell lines, which express high amounts of CD44 and are surrounded by hyaluronan containing pericellular matrices, were studied [46]. The function of microvascular endothelial cell CD44 was studied by pre-treatment of TIME cells with Hermes-1 antibodies that could block its interaction with either peritumoral or hyaluronan synthesized by TIME cells. Interestingly, an about 50% reduction in the adhesive capacity of breast cancer cell lines was detected already after 15 min and was sustained for up to 4 h (Figure 2A). Thus, CD44 expressed by TIME cells is crucial for regulation of both the early and late adhesion of breast cancer cells.

We then investigated the role of hyaluronan surrounding the MDA-MB-231 and MDA-MB-231-BM cells for their adhesion to TIME cells. Breast cancer cells were pre-treated or not with *Streptomyces* hyaluronidase, in order to remove the pericellular hyaluronan, before their seeding on TIME cell monolayers. Such treatment reduced their adhesiveness at early time points (15–60 min) by about 40–50% compared to untreated cells. However, the relevance of peri-tumoral hyaluronan for the adherence of breast cancer cells to microvascular endothelial cell monolayers was less pronounced at the later stages of adhesion i.e. at 4 h (Figure 2B), suggesting that peritumoral hyaluronan can mediate the “rolling phase” contact of breast cancer cells with the endothelium. Thus, hyaluronan binding to CD44 affects the adhesiveness of breast cancer cells to microvascular endothelial cells.

### Suppression of HYAL2 or CD44 differentially modulates the structure of endothelial cell tube-like formations

**Figure 3:**
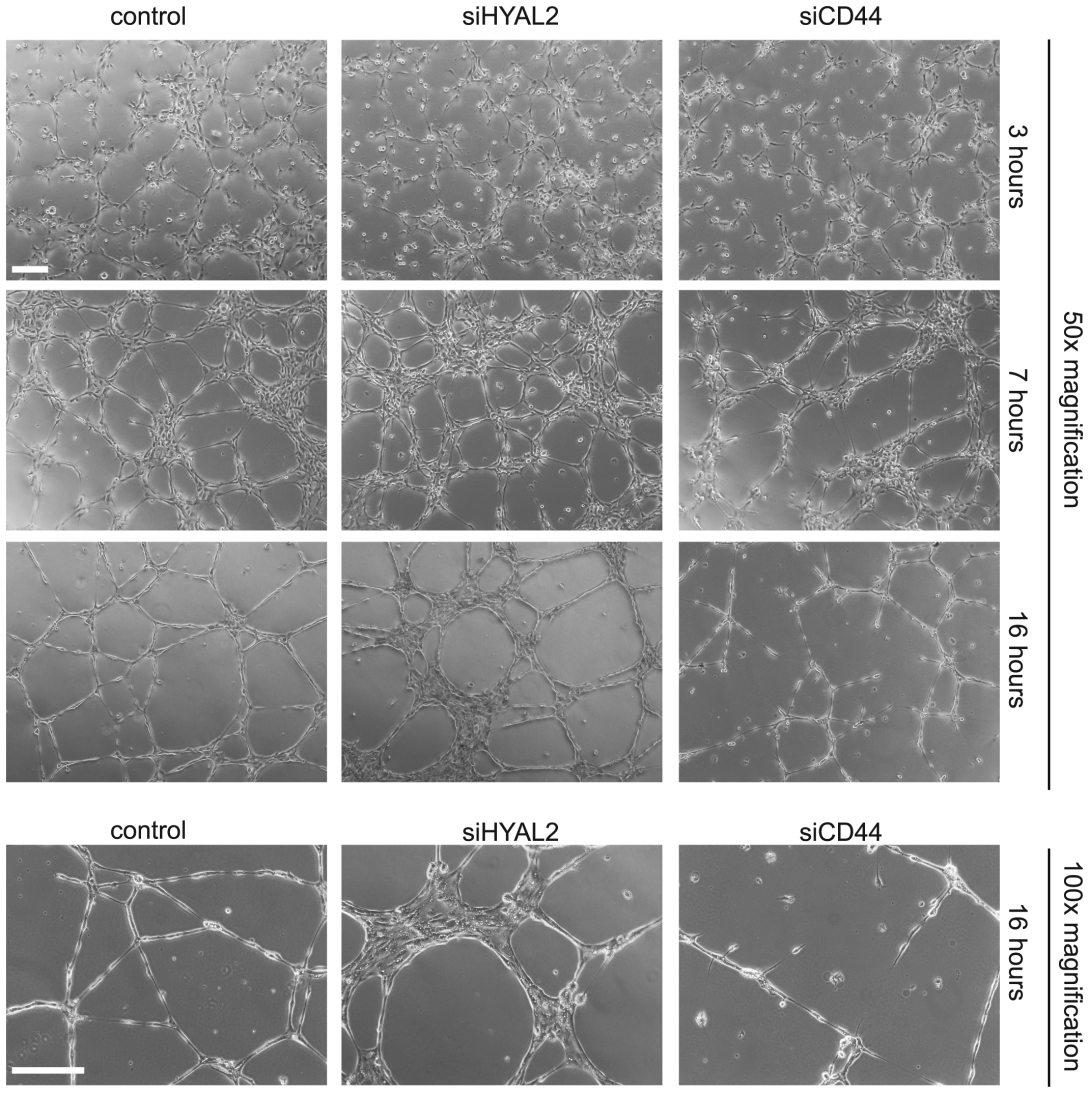
Knockdown of HYAL2 or CD44 impairs tubulogenesis. TIME cells were transfected with scrambled control siRNA or siRNAs for HYAL2 or CD44 for 24 h, before seeded onto Matrigel. Phase contrast overview photos, using a Zeiss Axiovert40 microscope, were taken at the indicated time points. A representative experiment out of three experiments performed in duplicates is depicted. Scale bar, 200 µm.

Previous studies revealed that HYAL-mediated hyaluronan fragmentation induces the formation of new blood vessels in chorioallantoic membrane and tubular structures in 3D collagen gels [13], [15], [16], [17]. However, the mechanism involved has not been extensively studied. The predominant high expressions of HYAL2 and CD44 mRNAs in TIME cells (Figure 1) prompted us to investigate their role during tubular morphogenesis. We investigated the effects of HYAL2 and CD44 silencing on the capability of endothelial cells to differentiate to vessel-like structures. As shown in Figure 3, microvascular endothelial cells expressing both HYAL2 and CD44 differentiated to form a network of tubular structures in a time-dependent manner. Within 3 h after seeding, cells elongated and connected with each other forming a network. At 7 h of culture, tubular-like structures in the network were apparent and the process peaked at 16 h after seeding. Cells depleted of HYAL2 were unable to differentiate to a regular vessel-like network; rather, after 16 h of culture cell aggregates composed of two or more clustered cell layers, were observed. In contrast, CD44-depletion resulted in an immature network of tubular structures (Figure 3). Notably, knockdown of both CD44 and HYAL2 resulted in cell death before 16 h of culture on Matrigel (data not shown) and was not investigated further.

### Molecular profiling of CD44- and HYAL2-deficient microvascular endothelial cells undergoing tubulogenesis

**Figure 4:**
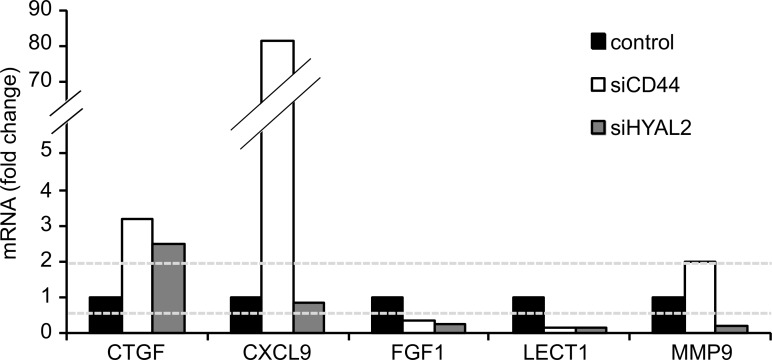
Gene expression by TIME cells undergoing tubulogenesis upon knockdown of HYAL2 or CD44. Microvascular endothelial cells transfected with siRNA for scrambled control, HYAL2 or CD44 were grown under differentiating conditions in Matrigel for 16-change lower than 0.5 were considered as downregulated, whereas genes with a fold-change above 2 were considered as upregulated.

To gain insights into the molecular mechanisms whereby CD44 and HYAL2 affect endothelial cell differentiation, we used a PCR Array to compare the induction of genes involved in angiogenesis of differentiating cells harvested from Matrigel, expressing or not expressing CD44 or HYAL2. The expression levels of genes in cells transfected with scrambled control siRNA were set to 1, and genes with fold-changes above 2 or below 0.5 were considered to be up- or downregulated, respectively. Whereas several genes in the PCR Array where affected upon suppression of CD44 or HYAL2 (Table S1), the connective tissue growth factor (CTGF) was up-regulated in HYAL2- or CD44-depleted cells (Figure 4). The matrix metalloproteinase 9 (MMP9) was slightly induced in CD44-depleted cells, whereas strongly suppressed in HYAL2-depleted cells, however, its expression in scrambled control siRNA transfected TIME cells was very low. Moreover, the fibroblast growth factor 1 (FGF1) and leukocyte cell-derived chemotaxin 1 (LECT1) were down-regulated after silencing of HYAL2 or CD44 compared to scrambled control transfected cells. Of particular interest was the induction of the chemokine (C-X-C) ligand 9 (CXCL9) in CD44-depleted cells, which was not seen in HYAL2-depleted cells.

### Silencing of CD44 in differentiating endothelial cells induces the expression of chemokine CXCL9, CXCL12 and their receptors

**Figure 5:**
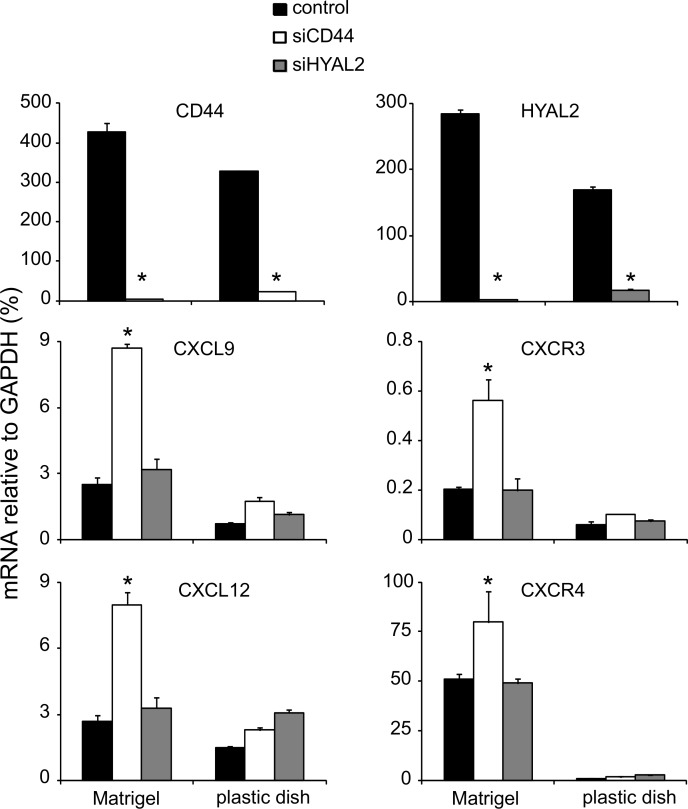
Gene expression levels of CXCL9 and CXCL12 and their receptors upon knockdown of CD44 or HYAL2 under proliferating and differentiating conditions. TIME cells transfected with scrambled control siRNA or siRNA against CD44 or HYAL2 were grown on Matrigel or on plastic dishes. RNA was extracted, reversely transcribed and subjected to real time PCR. Gene expression of the chemokines CXCL9 and CXCL12 and their receptors CXCR3 and CXCR4, respectively, were determined, as described in Materials and Methods. A representative experiment out of three performed in triplicates with similar results is shown ± SD.

Using real time PCR, we validated the significant up-regulation of CXCL9 mRNA in TIME cells undergoing morphogenesis and depleted of CD44. Such an induction of CXCL9 was not observed under proliferative conditions (Figure 5). CXCL9 signals via the receptor CXCR3, which as well was found to be up-regulated in differentiating, but not in proliferating, microvascular endothelial cells. Because there is a cross talk between CXCR3 and CXCR4 chemokine receptors [54], we investigated the expression levels of CXCR4 receptor and its ligand chemokine CXCL12 [55]. As shown in Figure 5, strong inductions of both CXCL12 and its receptor CXCR4 were detected under differentiating conditions in Matrigel, but not under proliferative conditions on solid surface, upon silencing of CD44. Notably, CXCR4 mRNA was expressed by TIME cells to more than 100-fold higher levels than CXCR3 mRNA under both proliferative and differentiating conditions. No induction of neither CXCL9 and CXCL12, nor their receptors CXCR3 and CXCR4, respectively, were observed in HYAL2-depleted cells (Figure 5).

### Effect of CD44 depletion on signaling pathways

**Figure 6:**
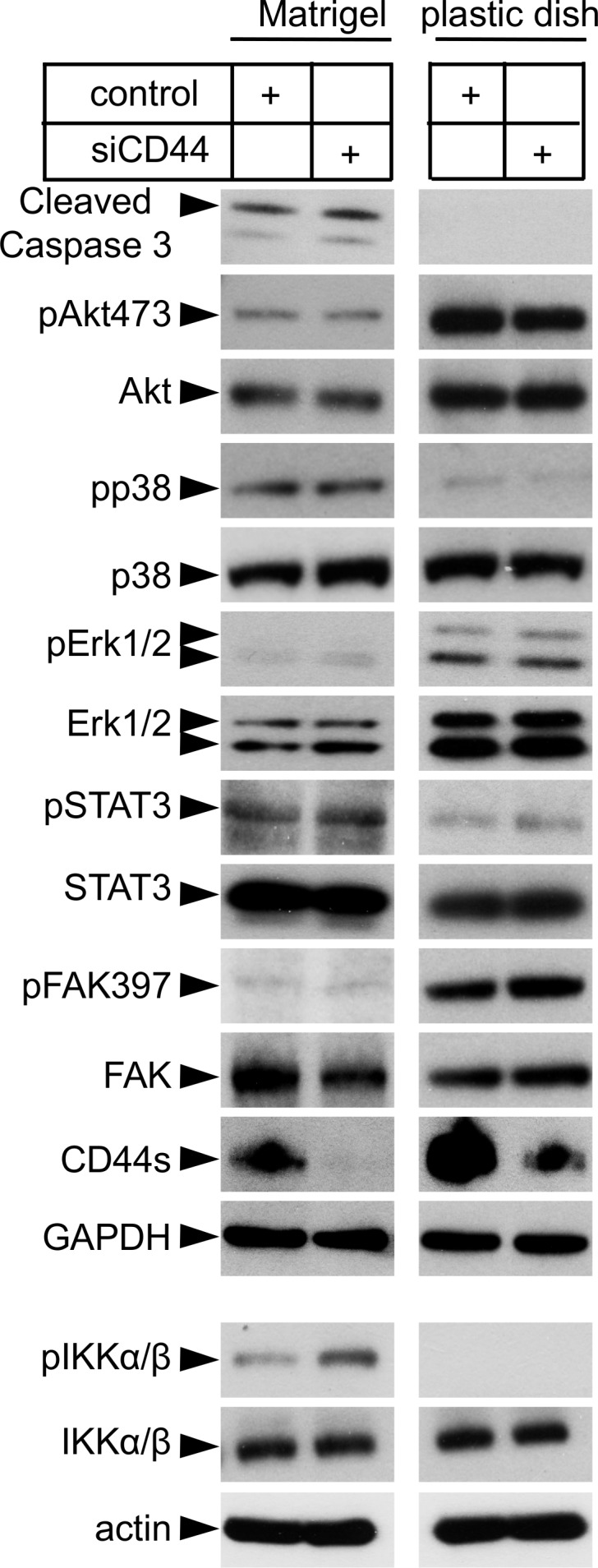
Effect of CD44 knockdown on the Erk MAPK pathway and NFκB target genes. Endothelial cells transfected with scrambled control siRNA or siRNA against CD44 were grown under differentiating or proliferating conditions and subjected to SDS-PAGE followed by immunoblotting for Cleaved Caspase 3 and the phosphorylated and total versions of proteins known to be involved in the Erk- and p38 MAPK-signaling, as well as STAT3, FAK and NFκB signaling. A representative experiment out of four experiments performed with similar results is shown ± SD.

The signaling mediated by chemokine receptors is both ligand- and cell-type dependent [54],[56],[57] and includes activation of the MEK-Erk and PI3K-Akt pathways. We studied the activation of signaling molecules after knock-down of CD44 under both proliferating and differentiating conditions. Upon knock-down of CD44 no induction in the apoptosis marker Cleaved Caspase 3 could be detected suggesting that the immature tubular structures formed (Figure 3) were not due to increased rate of apoptosis upon the loss of CD44. Furthermore, no significant differences could be detected in the levels of expression or activation of Akt, p38, Erk, STAT3 and FAK molecules (Figure 6).

**Figure 7:**
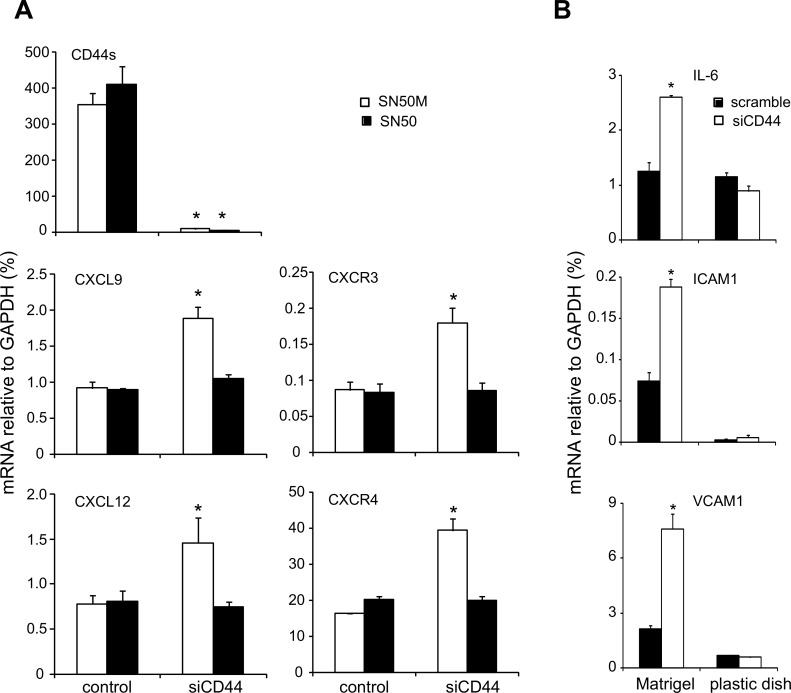
Effects of inhibition of NFκB translocation into the nucleus and CD44 silencing on NFκB target genes. (A) TIME cells were transfected with scrambled control siRNA or siRNA against CD44 for 24 h. Then, cells were pretreated for 1 h with 36 µM NFκB SN50 inhibitor or inactive control SN50M peptide, followed by culture into Matrigel for 16 h. RNA was extracted and the mRNA levels of CXCL9, CXCL12, CXCR3 and CXCR4 were determined via real time PCR, as described in Materials and Methods. (B) TIME cells expressing CD44 or not, were harvested after 16 h of culture into Matrigel or on plastic dish and RNA was extracted and analysed via real time PCR for the NFκB target genes IL-6, ICAM-1 and VCAM-1. A representative experiment out of three performed in triplicates with similar results is shown ± SD.

The genes encoding chemokine receptors and their ligands can be target genes for the binding of the nuclear transcription factor NFκB [57], [58]. The activation of NFκB depends on the activated IκB kinase (IKK) complex, which is composed of two catalytic subunits IKKβ and IKKβ [59]. As shown in Figure 6, the expression of phosphorylated IKK complex was high in CD44-depleted endothelial cells after 16 h of differentiation, indicating an active NFκB signaling pathway. To explore this finding further, we treated cells with the cell-permeable NFκB inhibitor SN50, which prevents the translocation of active NFκB to the nucleus, or with an inactive control SN50M peptide. Interestingly, treatment of CD44-depleted endothelial cells grown in Matrigel with the SN50 NFκB inhibitor reversed the upregulation of CXCL9, CXCR3, CXCL12 and CXCR4 induced by silencing of CD44 (Figure 7A). To further confirm the NFκB-mediated upregulation of CXCL9, CXCR3, CXCL12 and CXCR4, we examined the expression of other genes known to be upregulated by NFκB signaling, including interleukin 6 (IL-6), intercellular adhesion molecule 1 (ICAM-1) and vascular cell adhesion molecule 1 (VCAM-1); all these genes were up-regulated after CD44 knockdown (Figure 7B).

### CD44 is expressed at fusion and vacuole sites during microvascular endothelial cell tubulogenesis

**Figure 8:**
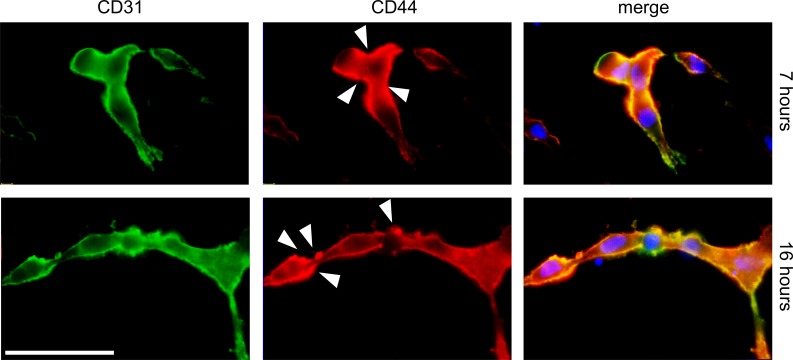
Detection of CD44 during tubulogenesis at early and late time points. TIME cells were cultured under differentiating conditions and the formed tubular structures were immunostained for CD44 (red) and the endothelial cell marker CD31 (green). The right picture shows the merge plus DAPI (blue). Arrows indicate CD44 expression at endothelial cell fusion sites. Their degree of tube formation was analyzed after 7 hours and 16 hours by microscopy. Scale bar, 10 µm.

To investigate the morphological changes during the tube assembly process in 3D culture conditions, cells were stained for CD31 and CD44. As shown in Figure 8, cells allowed to sprout for 7 h were fused and started to form tubular networks characterized by the expression of CD31 and CD44 at the plasma membrane. Notably, CD44 expression was predominantly seen at sites of plasma membrane fusion. After 16 h of culture, TIME cells were assembled into multicellular tubes; CD44 expression was seen at vacuole-like structures that appeared to have undergone fusion with the plasma membrane.

## Discussion

In this study we report that CD44 and HYAL2 are expressed at high levels in microvascular endothelial cell cultures. Functionally, these molecules act in a regulatory network including NFκB target genes in the regulation of TIME cell tubulogenesis. We show that CD44 and HYAL2 are required for tubulogenesis, and their repression impairs the formation of a regular vessel-like network. Most likely, the silencing of HYAL2 results in the accumulation of high molecular mass hyaluronan which sequestrated on the surface of TIME cells, via CD44 or HASes, bridges adjacent microvascular endothelial cells promoting cell-cell adhesion (Figure 3). Although hyaluronan oligosacharides are the primary mediators of hyaluronan-induced angiogenesis [15], [16], [17], [60], [61], it has been shown that high molecular mass hyaluronan can induce the migration of bovine aortic endothelial cells through the activation of Rho GTPases [62]. More recently, a study on human umbilical vein endothelial cells revealed that high molecular mass hyaluronan in a CXCL12-dependent signaling induces angiogenesis [63]. In contrast to hyaluronan oligosacharides that triggers signal transduction pathways [36], endothelial cells treated with high molecular mass hyaluronan induce only a weak protein kinase intracellular signaling. Furthermore, human endothelial cells, originating from microvessels or large arteries, differ in their ability to bind high molecular mass hyaluronan or hyaluronan fragments [64]. In addition, large vessel endothelium might differ functionally from small vessel endothelium. Hyaluronan binding to CD44 affects the adhesiveness of breast cancer cells, but also converts signals via CD44 resulting in regulation of gene expression such as the expression of chemokine genes (Figure 5).

Chemokines are small pro-inflammatory chemoattractant cytokines that promote leukocyte migration, and play key regulatory roles during embryogenesis, hematopoiesis, cancer and angiogenesis [65], [66]. Despite the critical role of chemokines in angiogensis, the molecular mechanisms underlying their effects are unclear [67]. The multiple transduction pathways activated by the crosstalk between chemokines receptors and their ligands is context-dependent, due to their promiscuous (several chemokines bind to one receptor) and pleiotropic (a chemokine binds to several receptors) character [56], [68]. Notably, positively charged chemokines can interact with the negatively charged cell surface or stromal proteoglycans and glycosaminoglycans, such as hyaluronan [69], [70]. It is possible that such chemokine-glycosaminoglycan interaction drives the formation of immobilized or haptotactic gradients and thereby modulates receptor activation and cellular responses. Chemokine-mediated chemotaxis is correlated with their ability to induce angiogenesis *in vivo* [43]. The observed increases in HAS1 and HAS2 mRNA levels under TIME cell differentiation (Figure 1B), suggest an active role of HASes and subsequently of hyaluronan during tubulogenesis. The slight increase in HYAL1 mRNA and the constitutive high expression of HYAL2 might further lead to the production of angiogenic hyaluronan fragments that through their interactions with CD44 (and constitutive secreted amounts of CXCL12) promotes angiogenesis (Figure 5). Interestingly, at inflammatory sites the local environment is enriched in reactive oxygen species and HYALs, which can depolymerize hyaluronan into oligosacharides that engage CD44 in endothelial tubulogenesis [16], [17], [39], [41]. In bronchial epithelial cells HYAL2 is induced in a p38 MAPK-dependent manner [71], [72].

Previous studies have suggested an interaction between hyaluronan-activated CD44 and CXCL12/CXCR4 signaling in induction of leukemia cell and human umbilical endothelial cell-polarization and subsequent migration [63], [73]. Ligand-induced CXCR4 activation promotes angiogenesis via stimulation of endothelial cell migration and proliferation, as well as VEGF production [43]. However, CXCR3 activated by its ligand CXCL9 suppresses the proliferation of microvascular ECs and exhibit an angiostatic activity [74]. Our studies demonstrate an inverse correlation between CD44 and the expression of the chemokines CXCL9 and CXCL12, and their receptors. The failure to form vessel-like structures upon suppresion of CD44 is associated with an NFκB-dependent upregulation of chemokines and their receptors in microvascular ECs studied (Figure 5 and 7). CD44 and other adhesion molecules are well known for their fine-tuning of signaling processes [33]. Notably, high and low molecular mass hyaluronan elicit differential signaling via CD44 leading to strengthening and disruption of contacts between endothelial cells, respectively [6].

The observations that CD44 is localized at plasma membrane vacuole-like fusion sites (Figure 8) and the inability of CD44-depleted TIME cells to form a tubular network, together with our previous finding that hyaluronan fragments initiate CD44-mediated tubulogenesis in a CXCL1-dependent manner [16], [17], supports key regulatory interdependent roles of hyaluronan binding to CD44 and chemokines in tubulogenesis. The important role of CD44 in TIME cell differentiation, does not exclude that other hyaluronan receptors such as RHAMM also affect TIME cell functions. Several angiogenic factors are heparin-binding proteins. The standard form of CD44 and most of its splice variants contain chondroitin sulphate polysaccharide chains. Only CD44v3 is decorated by heparin sulphate enabling it to bind growth and angiogenic factors including VEGF, bFGF and HGF. However, the CD44v6 has also been demonstrated to function as a co-receptor for receptor tyrosine kinase c-Met on epithelial cells [75], and to co-operate with VEGFR-2-mediated angiogenesis in endothelial cells [76]. The expression of standard isoform of CD44 dominates on TIME cells and most likely mediates the effects on angiogenesis, however, we knock-down all splice forms of CD44, therefore specific functions of the variant splice forms of CD44 cannot be excluded.

In summary, we have uncovered a novel mechanism where the expression of the hyaluronan receptor CD44 and the hyaluronidase HYAL2, in a coordinated fashion, regulate hyaluronan content in endothelial glycocalyx and affect CD44-mediated tubulogenesis by affecting the expressions of the cytokines CXCL9 and CXCL12 as well as their receptors.

## Supporting Information

Table S1 **Molecular profiling of CD44 and HYAL2 depleted TIME cells undergoing morphogenesis.** TIME cells were transfected with siRNA (scrambled control, HYAL2 and CD44) and grown under differentiating conditions as described in Materials and Methods. The fold-change of gene expression of the listed genes (scrambled control was arbitrarily set to 1) was quantified using an angiogenesis –specific RT^2^ Profiled PCR array.
(DOCX)
