# Supplementary material for: Knock-Down of CD44 Regulates Endothelial Cell Differentiation via NFκB-Mediated Chemokine Production
Source: PLoS One. 2014 Mar 10;9(3):e90921. doi: 10.1371/journal.pone.0090921 (PMC3948721; doi:10.1371/journal.pone.0090921)
Supplement: Table S1 — Molecular profiling of CD44 and HYAL2 depleted TIME cells undergoing morphogenesis. TIME cells were transfected with siRNA (scrambled control, HYAL2 and CD44) and grown under differentiating conditions as described in Materials and Methods. The fold-change of gene expression of the listed genes (scrambled control was arbitrarily set to 1) was quantified using an angiogenesis –specific RT2 Profiled PCR array. (DOCX) [file pone.0090921.s001.docx]

**Table S1.**

| **GeneBank** | **Symbol** | **Gene Name** | **Fold change** | |
| --- | --- | --- | --- | --- |
|  |  |  | **siHYAL2** | **siCD44** |
| NM_005163 | AKT1 | v-akt murine thymoma viral oncogene homolog 1 | 0.98 | 0.88 |
| NM_001145 | ANG | angiogenin, ribonuclease, RNase A family, 5 | 0.93 | 0.84 |
| NM_001147 | ANGPT2 | angiopoietin 2 | 1.23 | 0.95 |
| NM_001039667 | ANGPTL4 | angiopoietin-like 4 | 1.33 | 1.38 |
| NM_001150 | ANPEP | alanyl (membrane) aminopeptidase | 1.02 | 0.84 |
| NM_001702 | BAI1 | brain-specific angiogenesis inhibitor 1 | 0.85 | 0.92 |
| NM_002982 | CCL2 | chemokine (C-C motif) ligand 2 | 0.92 | 1.34 |
| NM_001795 | CDH5 | cadherin 5, type 2 (vascular endothelium) | 1.01 | 0.97 |
| NM_030582 | COL18A1 | collagen, type XVIII, alpha 1 | 0.97 | 0.68 |
| NM_000091 | COL4A3 | collagen, type IV, alpha 3 | 0.85 | 0.92 |
| NM_001901 | CTGF | connective tissue growth factor | 2.51 | 3.23 |
| NM_001511 | CXCL1 | chemokine (C-X-C motif) ligand 1 | 1.27 | 1.06 |
| NM_002994 | CXCL5 | chemokine (C-X-C motif) ligand 5 | 0.66 | 0.68 |
| NM_002993 | CXCL6 | chemokine (C-X-C motif) ligand 6 | 1.08 | 1.84 |
| NM_002416 | CXCL9 | chemokine (C-X-C motif) ligand 9 | 0.85 | 81.08 |
| NM_001955 | EDN1 | endothelin 1 | 1.08 | 1.13 |
| NM_182685 | EFNA1 | ephrin-A1 | 0.92 | 0.79 |
| NM_004093 | EFNB2 | ephrin-B2 | 0.92 | 0.78 |
| NM_000118 | ENG | endoglin | 1.01 | 1.00 |
| NM_004444 | EPHB4 | EPH receptor B4 | 1.00 | 0.89 |
| NM_004448 | ERBB2 | v-erb-b2 erythroblastic leukemia viral oncogene homolog 2 | 1.71 | 1.89 |
| NM_001993 | F3 | coagulation factor III (thromboplastin, tissue factor) | 0.96 | 1.02 |
| NM_000800 | FGF1 | fibroblast growth factor 1 | 0.24 | 0.35 |
| NM_002006 | FGF2 | fibroblast growth factor 2 | 1.26 | 0.89 |
| NM_000142 | FGFR3 | fibroblast growth factor receptor 3 | 0.55 | 1.07 |
| NM_004469 | FIGF | c-fos induced growth factor (vascular endothelial growth factor D) | 0.58 | 0.70 |
| NM_002019 | FLT1 | fms-related tyrosine kinase 1 (vascular endothelial growth factor/vascular permeability factor receptor) | 0.65 | 0.90 |
| NM_002026 | FN1 | fibronectin 1 | 1.05 | 0.76 |
| NM_001530 | HIF1A | hypoxia inducible factor 1, alpha subunit | 0.89 | 1.05 |
| NM_006665 | HPSE | heparanase | 0.99 | 1.02 |
| NM_002165 | ID1 | inhibitor of DNA binding 1 | 1.04 | 0.88 |
| NM_000619 | IFNG | interferon, gamma | 0.85 | 0.92 |
| NM_000618 | IGF1 | insulin-like growth factor 1 | 0.85 | 0.92 |
| NM_000576 | IL1B | interleukin 1, beta | 0.90 | 1.08 |
| NM_000600 | IL6 | interleukin 6 | 1.01 | 1.09 |
| NM_000584 | IL8 | interleukin 8 | 1.34 | 1.26 |
| NM_002210 | ITGAV | integrin, alpha V | 1.02 | 1.37 |
| NM_000212 | ITGB3 | integrin, beta 3 | 1.40 | 0.94 |
| NM_000214 | JAG1 | jagged 1 | 1.03 | 0.91 |
| NM_002253 | KDR | kinase insert domain receptor | 1.08 | 0.95 |
| NM_007015 | LECT1 | leukocyte cell derived chemotaxin 1 | 0.14 | 0.15 |
| NM_000230 | LEP | leptin | 0.72 | 1.77 |
| NM_002391 | MDK | midkine (neurite growth-promoting factor 2) | 1.21 | 1.34 |
| NM_004995 | MMP14 | matrix metallopeptidase 14 (membrane-inserted) | 1.22 | 0.87 |
| NM_004530 | MMP2 | matrix metallopeptidase 2 | 0.98 | 0.92 |
| NM_004994 | MMP9 | matrix metallopeptidase 9 | 0.22 | 1.98 |
| NM_000603 | NOS3 | nitric oxide synthase 3 (endothelial cell) | 0.98 | 0.78 |
| NM_004557 | NOTCH4 | notch 4 | 0.98 | 0.87 |
| NM_003873 | NRP1 | neuropilin 1 | 1.06 | 0.86 |
| NM_003872 | NRP2 | neuropilin 2 | 0.98 | 0.97 |
| NM_002607 | PDGFA | platelet-derived growth factor alpha polypeptide | 0.95 | 1.06 |
| NM_000442 | PECAM1 | platelet/endothelial cell adhesion molecule 1 | 0.93 | 1.27 |
| NM_002619 | PF4 | platelet factor 4 | 0.85 | 0.92 |
| NM_002632 | PGF | placental growth factor | 0.95 | 0.93 |
| NM_002658 | PLAU | plasminogen activator, urokinase | 1.03 | 1.03 |
| NM_000301 | PLG | plasminogen | 0.77 | 0.88 |
| NM_000962 | PTGS1 | prostaglandin-endoperoxide synthase 1 | 0.91 | 0.90 |
| NM_001400 | S1PR1 | sphingosine-1-phosphate receptor 1 | 1.04 | 1.03 |
| NM_000602 | SERPINE1 | serpin peptidase inhibitor, clade E, member 1 | 0.94 | 1.19 |
| NM_002615 | SERPINE2 | serpin peptidase inhibitor, clade F, member 1 | 0.75 | 0.88 |
| NM_021972 | SPHK1 | sphingosine kinase 1 | 1.01 | 0.91 |
| NM_000459 | TEK | tyrosine kinase, endothelial | 1.03 | 1.09 |
| NM_003236 | TGFA | transforming growth factor, alpha | 0.92 | 1.63 |
| NM_000660 | TGFB1 | transforming growth factor, beta 1 | 0.95 | 0.84 |
| NM_003238 | TGFB2 | transforming growth factor, beta 2 | 1.04 | 0.90 |
| NM_004612 | TGFBR1 | transforming growth factor, beta receptor 1 | 1.33 | 1.20 |
| NM_003246 | THBS1 | thrombospondin 1 | 0.85 | 0.97 |
| NM_005424 | TIE1 | tyrosine kinase with immunoglobulin-like and EGF-like domains 1 | 0.97 | 0.85 |
| NM_003254 | TIMP1 | metallopeptidase inhibitor 1 | 1.00 | 1.12 |
| NM_003255 | TIMP2 | metallopeptidase inhibitor 2 | 1.00 | 0.92 |
| NM_000362 | TIMP3 | metallopeptidase inhibitor 3 | 0.85 | 0.92 |
| NM_000594 | TNF | tumor necrosis factor | 1.15 | 1.47 |
| NM_001953 | TYMP | thymidine phosphorylase | 1.32 | 1.30 |
| NM_003376 | VEGFA | vascular endothelial growth factor A | 1.03 | 0.96 |
| NM_003377 | VEGFB | vascular endothelial growth factor B | 0.99 | 0.88 |
| NM_005429 | VEGFC | vascular endothelial growth factor C | 0.93 | 1.18 |
